# Predictive factors for oocyte retrieval failure in controlled ovarian hyperstimulation protocols: a retrospective observational cohort study

**DOI:** 10.1186/s12958-015-0052-x

**Published:** 2015-06-02

**Authors:** Ayumi Hasegawa, Toshifumi Takahashi, Hideki Igarashi, Mitsuyoshi Amita, Jun Matsukawa, Satoru Nagase

**Affiliations:** Department of Obstetrics and Gynecology, Yamagata University Faculty of Medicine, Yamagata, 990-9585 Japan

**Keywords:** Assisted reproductive technology, Oocyte retrieval, Empty follicle syndrome, Controlled ovarian hyperstimulation, Gonadotropin-releasing hormone agonist, Gonadotropin-releasing hormone antagonist

## Abstract

**Background:**

Oocyte retrieval failure following an ovarian hyperstimulation protocol is uncommon in assisted reproductive technology (ART) programs. We analyzed the predictive factors for oocyte retrieval failure following controlled ovarian hyperstimulation (COH) with gonadotropin-releasing hormone (GnRH) agonist and GnRH antagonist protocols in ART programs.

**Methods:**

This study was a retrospective cohort observational study. In total, 744 cycles from 361 patients who underwent controlled ovarian hyperstimulation with GnRH agonist long protocol or antagonist protocol were analyzed. Treatment cycles with oocyte retrieval failure and with one or more oocytes retrieved were compared to determine predictive factors for oocyte retrieval failure using univariate and multilevel multivariate logistic regression analyses.

**Results:**

Oocyte retrieval failure occurred in 38 cycles (5.1 %). The oocyte retrieval failure rate of the GnRH antagonist protocol (8.1 %) was significantly higher than that of the GnRH agonist long protocol (3.7 %). On multilevel multivariate logistic analysis, cycles with GnRH antagonist protocol (odds ratio [OR] 3.06, 95 % confidence interval [CI] 1.05–8.96), estradiol level on the day of human chorionic gonadotropin (hCG) injection (OR 0.997, 95 % CI 0.996–0.998), and luteinizing hormone (LH) level on the day of hCG injection (OR 1.19, 95 % CI 1.06–1.33) were independent predictive factors for oocyte retrieval failure. The efficacy of estradiol and LH levels on the day of hCG injection for predicting oocyte retrieval failure was evaluated using receiver operating characteristic curves. In all cycles, the areas under the curve (AUCs) for estradiol and LH were 0.84 and 0.63, respectively, for all cycles; 0.84 and 0.52, respectively, for cycles with GnRH agonist long protocol; and 0.81 and 0.82, respectively, for cycles with GnRH antagonist protocol.

**Conclusions:**

Our results suggest that in cycles with GnRH antagonist protocol, the levels of estradiol and LH on the day of hCG injection might be predictive factors for oocyte retrieval failure. This relationship may provide useful information to both patients and physicians for developing better COH protocols in ART programs.

## Background

Assisted reproductive technology (ART) has been widely used for infertility treatment [1]. Acquisition of oocytes is the first step towards successful outcomes in an ART program. Ovarian hyperstimulation with gonadotropin-releasing hormone (GnRH) agonist and GnRH antagonist protocols is used to maximize the number of retrieved oocytes [2].

Oocyte retrieval failure, i.e., zero oocytes retrieved following an ovarian hyperstimulation protocol, is uncommon in ART programs [3] and referred to as “empty follicle syndrome” [3, 4]. The incidence of oocyte retrieval failure, involving a minimum ovarian hyperstimulation protocol with clomiphene citrate, ranges from 0.045 % to 7 % [3]. It may cause substantial stress and anxiety for both patients and physicians. However, the etiology of oocyte retrieval failure remains unknown.

In the present study, we analyzed the predictive factors for oocyte retrieval failure following controlled ovarian hyperstimulation with GnRH agonist and GnRH antagonist protocols in ART programs.

## Methods

This study was a retrospective cohort observational study. A total of 744 cycles from 361 patients who underwent *in vitro* fertilization (IVF) and intracytoplasmic sperm injection (ICSI) programs in the period from November 2006 to November 2014 at Yamagata University Hospital, Yamagata, Japan, were analyzed. The Yamagata University Ethical Committee on human subjects approved the present study, and written informed consent was obtained from all patients.

### Controlled ovarian hyperstimulation and oocyte retrieval

All patients underwent controlled ovarian hyperstimulation (COH) by daily injections of human menopausal gonadotropin or recombinant follicle-stimulating hormone (FSH) and pituitary desensitization following a GnRH agonist long protocol or GnRH antagonist protocol. Cycle monitoring was carried out using transvaginal sonography. In the GnRH agonist long protocol, the patients received a GnRH agonist (Suprecure nasal spray, 600 or 900 μg daily, Mochida, Tokyo, Japan) from the mid-luteal phase of the previous cycle to the day of human chorionic gonadotropin (hCG) injection. In the GnRH antagonist protocol, the patients received a GnRH antagonist (Setrotide, 0.25 mg daily, Merck Serono, Tokyo, Japan), which was administered when the leading follicle was 13 to 14 mm in a diameter or on cycle day 8 and continued until the day of hCG injection. Cumulus oocyte complexes (COCs) were aspirated without flushing 36 h after hCG injection using an 18- or 19-gauge needle guided by transvaginal ultrasonography. The collected COCs were counted and subsequently inseminated using either conventional IVF or ICSI.

### Hormone assays

Hormone measurements were performed on the day of hCG injection. Hormone concentrations were quantified using commercially available immunoassay kits. Luteinizing hormone (LH), FSH, and prolactin (PRL) were measured using an electrochemiluminescence immunoassay (ECLusys reagent LH, FSH, PRL kit; Roche Diagnostics, Inc., Tokyo, Japan). Estradiol and progesterone levels were measured using a chemiluminescence immunoassay (Architect estradiol and progesterone kit; Abbott Japan, Inc., Tokyo, Japan). Reliability criteria for all assays were established. The interassay coefficient of variation was 3.3 % for estradiol and 7.9 % for progesterone. The intraassay coefficient of variation was 5.2 % for estradiol and 7.2 % for progesterone. All samples were assayed in duplicate.

### Statistical analysis

We compared various possible factors affecting oocyte retrieval between patients with zero oocytes retrieved and those from whom one or more oocytes were retrieved. Data are presented as mean ± SD if a normal distribution was expected; otherwise, median and range were used. In univariate analysis, differences in nominal variables between the groups were compared using the *χ*^2^ test, unless the expected frequency was < 5, in which case Fisher’s exact probability test was used. Continuous variables were analyzed using nonparametric Mann–Whitney *U* test. In the multivariate analysis, multilevel multivariate logistic regression models were used to determine the independent prognostic factors for oocyte retrieval failure. The first level was defined as the cycle and the second level was defined as the patient. This approach permitted analyses at the cycle level while adjusting for within-patient correlations [5]. The area under the receiver operating characteristic (ROC) curve was used to assess the discriminative ability of the logistic models. All statistical analyses were performed using Stata software version 13.1 (Stata Corp LP, College Station, TX, USA). All tests for significance were two-tailed, and significance was defined as *p* < 0.05.

## Results

The clinical characteristics of the patients are summarized in Table 1. The results of the univariate analyses of cycles with zero oocytes retrieved and cycles with one or more oocytes retrieved are shown in Table 2. Zero oocytes were retrieved in 38 cycles (5.1 % of cycles). The number of patients with zero oocytes retrieved was 34 (9.4 % of patients), because four patients experienced repeated oocyte failure. Both age and parity of the cycles with zero oocytes retrieved were significantly higher than those of the cycles with one or more oocytes retrieved. The rate of oocyte retrieval failure in cycles with GnRH antagonist protocol (8.1 %) was significantly higher than that in cycles with GnRH agonist long protocol (3.7 %). Levels of FSH and LH were significantly higher in cycles with zero oocytes retrieved than in those with one or more oocytes retrieved. Levels of PRL, estradiol, and progesterone were significantly lower in cycles with zero oocytes retrieved than in those with one or more oocytes retrieved. The number of follicles over 15.5 mm on the day of hCG injection was significantly lower in cycles with zero oocytes retrieved than in those with one or more oocytes retrieved.

**Table 1:** Patient characteristics

No. of cycles, n	744
No. of patients, n	361
Age, years	37 (23–46)^a^
Gravida per patient, n	0 (0–7)^a^
Parity per patient, n	0 (0–4)^a^
BMI, kg/m^2^	21 (16–36)^a^
Infertility period, years	6 (0–18)^a^
Previous treatment cycles, n	2 (1–16)^a^
Cycles with GnRH agonist long/all cycles (%)	509/744 (68.4)
Cycles with GnRH antagonist/all cycles (%)	235/744 (31.6)
Duration of hMG/rFSH, days	11 (7–21)^a^
Dose of hMG/rFSH, IU	1425 (150–4800)^a^
Hormone levels^b^	
FSH, mIU/ml	13.5 (3.2–113.5)^a^
LH, mIU/ml	2.4 (0.1–45.3)^a^
PRL, ng/ml	30.4 (0.6–314)^a^
Estradiol, pg/ml	1633 (62–15,768)^a^
Progesterone, ng/ml	0.75 (0.05–10.9)^a^
Endometrial thickness^b^, mm	10.9 (4.6–21.2)^a^
No. of follicles over 15.5 mm^b^, n	4 (1–16)^a^

^a^Median (range). ^b^Values on the day of hCG injection. BMI: body mass index; GnRH: gonadotropin-releasing hormone; hMG: human menopausal gonadotropin; rFSH: recombinant follicle stimulating hormone; IU: international unit; LH: luteinizing hormone; PRL: prolactin; hCG: human chorionic gonadotropin

**Table 2:** Univariate analysis of variables in the cycles with one or more oocytes retrieved and zero oocytes retrieved

	Oocyte retrieval (+)	Oocyte retrieval (−)	*P* value
No. of cycles/all cycles (%)	706/744 (94.9)	38/744 (5.1)	–
Age, years	37 (23–46)^a^	39 (31–46)^a^	0.01
Gravida per patient, n	0 (0–7)^a^	0 (0–6)^a^	0.25
Parity per patient, n	0 (0–3)^a^	0 (0–4)^a^	0.02
BMI, kg/m^2^	21 (16–36)^a^	21 (18–31)^a^	0.42
Infertility period, years	6 (0–18)^a^	7 (0–16)^a^	0.09
Previous treatment cycles, n	2 (1–16)^a^	2 (1–14)^a^	0.87
No. of cycles with GnRH agonist long/no. of cycles (%)	190/706 (69.4)	19/38 (50.0)	0.02
No. of cycles with GnRH antagonist/no. of cycles/ (%)	216/706 (30.6)	19/38 (50.0)	0.02
Duration of hMG/rFSH, days	11 (7–21)^a^	12 (8–20)^a^	0.49
Dose of hMG/rFSH, IU	1425 (150–4800)^a^	1500 (900–4800)^a^	0.20
Hormone levels^b^			
FSH, mIU/ml	13.4 (3.2–113.5)^a^	16.8 (7.5–43.5)^a^	<0.001
LH, mIU/ml	2.3 (0.1–26.8)^a^	3.8 (0.1–45.3)^a^	0.006
PRL, ng/ml	30.7 (0.6–314)^a^	21.3 (9.3–56.8)^a^	<0.001
Estradiol, pg/ml	1717 (88–15,768)^a^	633 (62–1970)^a^	<0.001
Progesterone, ng/ml	0.77 (0.05–10.9)^a^	0.51 (0.1–6.48)^a^	0.02
Endometrial thickness^b^, mm	10.9 (4.6–21.2)^a^	10.3 (6.2–17.2)^a^	0.51
No. of follicles over 15.5 mm^b^, n	4 (1–16)^a^	2 (1–6)^a^	<0.001

^a^Median (range). ^b^Values on the day of hCG injection. BMI: body mass index; GnRH: gonadotropin-releasing hormone; hMG: human menopausal gonadotropin; rFSH: recombinant follicle stimulating hormone; IU: international unit; LH, luteinizing hormone; PRL, prolactin; hCG: human chorionic gonadotropin

**Fig. 1:**
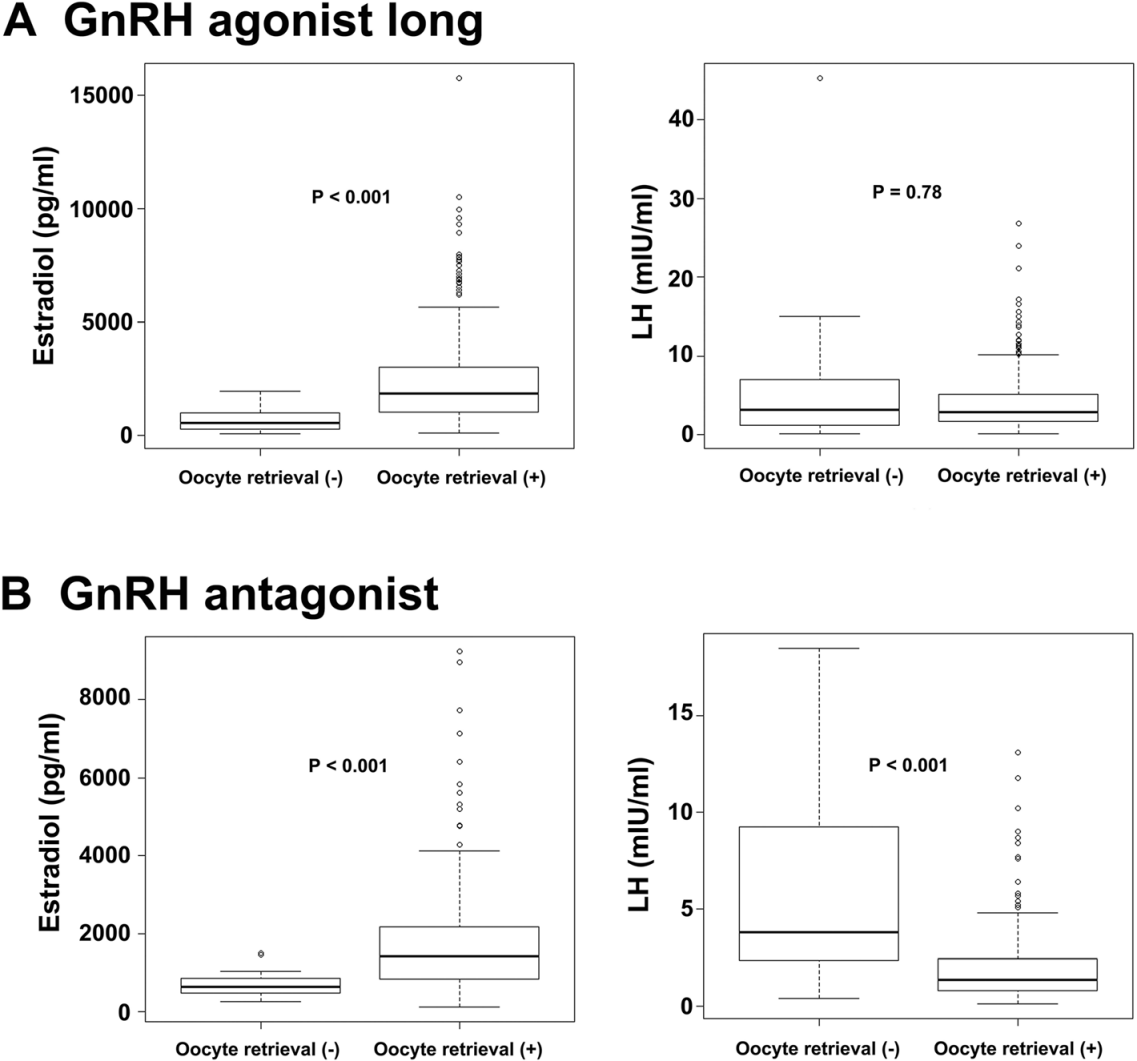
Levels of estradiol and LH on the day of hCG injection in patients who underwent GnRH agonist long and GnRH antagonist protocols in cycles with zero and one or more oocytes retrieved. **a**. levels of estradiol and LH on the day of hCG injection in cycles with GnRH agonist long protocol. **b**. levels of estradiol and LH on the day of hCG injection in cycles with GnRH antagonist protocol

On multilevel multivariate logistic analysis, GnRH antagonist protocol (odds ratio [OR] 3.06, 95 % confidence interval [CI] 1.05­8.96, *P* = 0.04), estradiol level on the day of hCG injection (OR 0.997, 95 % CI 0.996­0.998, *P* = 0.001), and LH level on the day of hCG injection (OR 1.19, 95 % CI 1.06­1.33, *P* = 0.003) were independent predictive factors for oocyte retrieval failure (Table 3). As these parameters appeared important for predicting oocyte retrieval failure, we compared the levels of estradiol and LH in cycles with GnRH agonist long and GnRH antagonist protocols (Fig. 1). Although the median level of estradiol on the day of hCG injection in cycles with zero oocytes retrieved was significantly lower than that in cycles with one or more oocytes retrieved, the median levels of LH on the day of hCG injection did not significantly differ between cycles with zero and one or more oocytes retrieved in cycles with GnRH agonist long protocol (Fig. 1a). By contrast, whereas the level of estradiol on the day of hCG injection in the cycles with zero oocytes retrieved was significantly lower than that in cycles with one more oocytes retrieved, the level of LH on the day of hCG injection in the cycles with zero oocytes retrieved was significantly higher than that in the cycles with one or more oocytes retrieved in cycles with GnRH antagonist protocol (Fig. 1b).

**Table 3:** Multilevel multivariate logistic analysis for oocyte retrieval failure

Variables	Odds ratio	95 % confidence interval	*P* value
Cycles with GnRH antagonist protocol	3.06	1.05–8.96	0.04
Estradiol on the day of hCG injection	0.997	0.996–0.998	0.0001
LH on the day of hCG injection	1.19	1.06–1.33	0.003

GnRH: gonadotropin releasing hormone; hCG: human chorionic gonadotropin; LH: luteinizing hormone

**Fig. 2:**
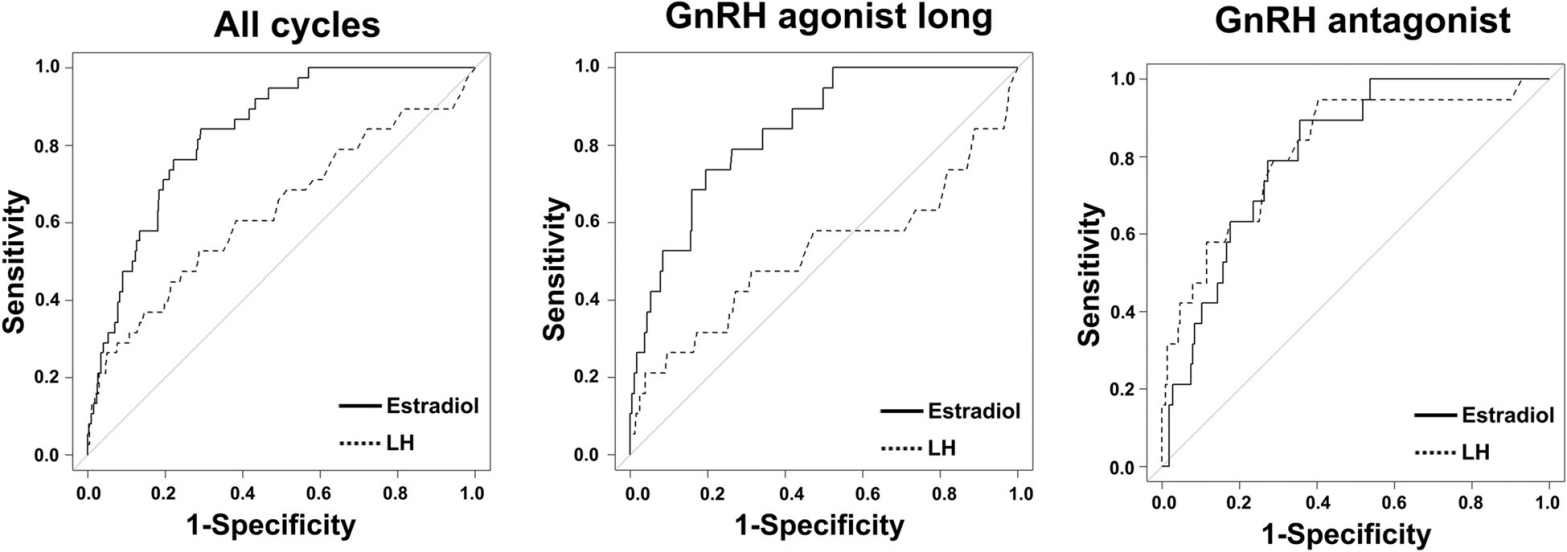
Receiver operating characteristic (ROC) curves of estradiol and LH on the day of hCG injection. ROC curves of estradiol and LH on the day of hCG injection in the cycles with all, GnRH agonist long, and GnRH antagonist protocols for predicting oocyte retrieval failure

Next, we validated the efficacy of the levels of estradiol and LH on the day of hCG injection in predicting the possibility of oocyte retrieval failure. Figure 2 shows the ROCs of estradiol and LH for the prediction of oocyte retrieval failure. In all cycles, the areas under the curve (AUCs) for estradiol and LH were 0.84 and 0.63, respectively. In cycles with GnRH agonist long protocol, the AUCs for estradiol and LH were 0.84 and 0.52, respectively. In cycles with GnRH antagonist protocol, the AUCs for estradiol and LH were 0.81 and 0.82, respectively. Table 4 summarizes the sensitivities, specificities, and positive and negative predictive values for predicting oocyte retrieval failure at various thresholds for estradiol and LH levels in all cycles, cycles with GnRH agonist long protocol, and cycles with GnRH antagonist protocol.

**Table 4:** Predictive efficacy of estradiol and LH levels on the day of hCG injection for oocyte retrieval failure

Type of COH	Threshold value	Sensitivity	Specificity	PPV	NPV
All cycles	Estradiol 1080 pg/ml	0.71	0.84	0.19	0.98
	LH 3.8 mIU/ml	0.71	0.53	0.07	0.97
Cycles with GnRH agonist long	Estradiol 887 pg/ml	0.81	0.74	0.14	0.99
	LH 10.9 mIU/ml	0.96	0.21	0.06	0.99
Cycles with GnRH antagonist	Estradiol 1040 pg/ml	0.64	0.90	0.24	0.98
	LH 1.7 mIU/ml	0.60	0.95	0.37	0.98

COH: controlled ovarian hyperstimulation; PPV: positive predictive value; NPV: negative predictive value; LH: luteinizing hormone; GnRH: gonadotropin-releasing hormone

## Discussion

We evaluated the predictive factors for oocyte retrieval failure with controlled ovarian hyperstimulation protocols in ART treatment cycles. The independent predictive factors for oocyte retrieval failure on multilevel multivariate analysis were use of the GnRH antagonist protocol and levels of estradiol and LH on the day of hCG injection. Analyses of ROCs revealed that estradiol level was more predictive of oocyte retrieval failure than LH level on the day of hCG injection in all treatment cycles, whereas estradiol and LH levels demonstrated similar predictive power for oocyte retrieval failure in cycles with GnRH antagonist protocol.

In the present study, we found that the odds ratio of oocyte retrieval failure with the GnRH antagonist protocol was three times that of the GnRH agonist long protocol. There have been no previous studies comparing types of COH in cycles with oocyte retrieval failure. The number of oocytes retrieved in cycles with GnRH antagonist protocol tends to lower than that in cycles with GnRH agonist long protocol [6, 7]. The etiology of the higher incidence of oocyte retrieval failure in cycles with GnRH antagonist protocol remains unclear. As the level of LH on the day of hCG injection was significantly higher in cycles with oocyte retrieval failure, insufficient LH suppression in the GnRH antagonist protocol might be a cause of oocyte retrieval failure.

A lower level of estradiol demonstrated positive predictive value for oocyte retrieval failure. Baum et al. reported that lower estradiol level on the day of hCG injection was a risk factor for oocyte retrieval failure in ART treatment cycles with controlled ovarian hyperstimulation protocols [8]. In the present study, the median level of estradiol in cycles with oocyte retrieval failure was three times greater than that in cycles with one or more oocytes retrieved. Previous studies also reported that lower estradiol level on the day of hCG injection was a risk factor for oocyte retrieval failure in ART treatment cycles with COH [9, 10]. The etiology of lower estradiol level on the day of hCG injection might be poor follicle development. In fact, we observed a lower number of developing follicles in cycles with oocyte retrieval failure compared to cycles with one or more oocytes retrieved.

Higher LH level on the day of hCG injection was also a positive predictor for oocyte retrieval failure. Choi et al. reported that the cancellation rate of ART treatment cycles was greater in cases of higher LH levels on the day of hCG injection than in cases of lower LH levels [11]. Premature LH surge results in cancellation of IVF treatment cycles or developmental arrest of oocytes because of early luteinization of immature follicles before oocyte retrieval [12–14]. Taken together, these results suggest that insufficient LH suppression during COH and fewer developing follicles might be a cause of oocyte retrieval failure.

Several candidates for the etiology of oocyte retrieval failure in ART treatment cycles have been proposed. First, the low bioavailability of hCG is one possible etiology of oocyte retrieval failure [15–17]. Zegers-Hochschild et al. reported that low bioavailability of hCG may be linked to intrinsic defects in the *in vivo* biological activity of some batches of hCG [15]. In the present study, the patients received hCG purchased from the same company, whose batches may have differed during the study period. Therefore, problems with the hCG drug might be a cause of oocyte retrieval failure. Reduced follicle development during COH is another possible etiology of oocyte retrieval failure [18]. Patients with a poor response to COH are vulnerable to oocyte retrieval failure [3, 7–9, 18]. These patients are considered to have a diminished ovarian reserve mainly due to ovarian aging [3, 9, 10]. In the present study, patient age was significantly higher in cycles with oocyte retrieval failure than those in which one or more oocytes were retrieved. Because we did not measure FSH levels at the early follicular phase or anti-Müllerian hormone, it is possible that patients with oocyte retrieval failure were subject to diminished ovarian reserve. The final possible etiology of oocyte retrieval failure is human error, such as a missed and/or incorrect dose of hCG injection [18–20]. We did not exclude possible cases of oocyte retrieval failure caused by human errors, such as a missed and/or incorrect dose of hCG injection.

When interpreting our results, the strengths and limitations of our study must be considered in light of its retrospective cohort design. There were potential confounding factors that should be acknowledged. In the present study, in univariate analysis, the patients’ age and parity were significantly higher in the cycles with zero oocytes retrieved than those of the cycles with one or more oocytes retrieved. To eliminate confounding factors, we applied multilevel multivariate analysis to determine the predictive factors for oocyte retrieval failure. As a result, age and parity were eliminated as candidate predictive factors for oocyte retrieval failure. However, because of its small sample size, the findings of the current study are vulnerable to type II error [21]. A larger-scale study is needed to evaluate the predictive factors for oocyte retrieval failure with COH in ART treatment cycles.

## Conclusions

Our results suggest that GnRH antagonist protocol, lower level of estradiol, and higher level of LH on the day of hCG injection might be positive predictors for oocyte retrieval failure. These findings provide useful information to both patients and physicians for developing better COH protocols in ART programs.

## Abbreviations

AUC: Area under the curve
ART: Assisted reproductive technology
CI: Confidence interval
COC: Cumulus oocyte complex
COH: Controlled ovarian hyperstimulation
FSH: Follicle stimulating hormone
GnRH: Gonadotropin-releasing hormone
hCG: Human chorionic gonadotropin
ICSI: Intracytoplasmic sperm injection
IVF: *In vitro* fertilization
LH: Luteinizing hormone
OR: Odds ratio
ROC: Receiver operating characteristic
